# Brain Sensory Organs of the Ascidian *Ciona robusta*: Structure, Function and Developmental Mechanisms

**DOI:** 10.3389/fcell.2021.701779

**Published:** 2021-09-06

**Authors:** Paola Olivo, Antonio Palladino, Filomena Ristoratore, Antonietta Spagnuolo

**Affiliations:** Department of Biology and Evolution of Marine Organisms, Stazione Zoologica Anton Dohrn, Napoli, Italy

**Keywords:** evolution, ascidians, pigmented cells, photoreceptor cells, molgula

## Abstract

During evolution, new characters are designed by modifying pre-existing structures already present in ancient organisms. In this perspective, the Central Nervous System (CNS) of ascidian larva offers a good opportunity to analyze a complex phenomenon with a simplified approach. As sister group of vertebrates, ascidian tadpole larva exhibits a dorsal CNS, made up of only about 330 cells distributed into the anterior sensory brain vesicle (BV), connected to the motor ganglion (MG) and a caudal nerve cord (CNC) in the tail. Low number of cells does not mean, however, low complexity. The larval brain contains 177 neurons, for which a documented synaptic connectome is now available, and two pigmented organs, the otolith and the ocellus, controlling larval swimming behavior. The otolith is involved in gravity perception and the ocellus in light perception. Here, we specifically review the studies focused on the development of the building blocks of ascidians pigmented sensory organs, namely pigment cells and photoreceptor cells. We focus on what it is known, up to now, on the molecular bases of specification and differentiation of both lineages, on the function of these organs after larval hatching during pre-settlement period, and on the most cutting-edge technologies, like single cell RNAseq and genome editing CRISPR/CAS9, that, adapted and applied to *Ciona* embryos, are increasingly enhancing the tractability of *Ciona* for developmental studies, including pigmented organs formation.

## Introduction

The sea squirt *Ciona intestinalis* represents a suitable model system for studying the transcriptional regulatory mechanisms exploited during chordate development (Satoh et al., 2003), due to its phylogenetic position (belonging to chordates as sister group of vertebrates) and to a number of peculiar features. These include the rapid embryogenesis as well as the small size of its larval stage (∼2,500 cells when fully developed) showing only six tissue types, as notochord, muscle, epidermis, endoderm, mesenchyme and nervous system together with a tunic, made of cellulose like material that covers the entire body of both, larva and adult, hence the name tunicates. *Ciona* is suitable for genetic studies since its small and compact genome (160 Mb and contains ∼15,500 genes) (Dehal et al., 2002) often contains single copy of genes present in multiple copies in the genome of vertebrates. This characteristic permits to reveal the function of the genes involved in fundamental steps of the developmental programs in the lineage of chordates. Furthermore, the simplicity of *Ciona* larval structures, in particular of its Central Nervous System (CNS), permits to depict the genetic programs adopted by a single blastomere in order to build up specific structures, tissues, organs present at the larval stage. This simplicity is undoubtedly a huge advantage compared to the complexity of vertebrates. In this context, the ascidian pigmented sensory organs represent, for more than two decades, a fertile ground for deep studies aimed at revealing their structure and function and deciphering the molecular mechanism leading to their development since the gastrula stage.

Inside ascidian group, recently, two cryptic species of *Ciona intestinalis*, types A and B (Caputi et al., 2007; Sato et al., 2012; Roux et al., 2013; Bouchemousse et al., 2016) have been described and proposed to be renamed, based on a taxonomic study (Brunetti et al., 2015), as *Ciona robusta* (*Ciona intestinalis* type A) and *Ciona intestinalis* (*Ciona intestinalis* type B). For the sake of simplicity and to avoid confusion, in this review the general term *Ciona* will be used for both species.

## Structure and Functions of Ascidian Pigmented Sensory Organs

In the tadpole larva of *Ciona*, the CNS is formed, from the anterior to the posterior, by three structures: the brain vesicle (or sensory vesicle), the visceral ganglion and the caudal nerve cord (Katz, 1983; Nicol and Meinertzhagen, 1991; Meinertzhagen et al., 2004).

**FIGURE 1:**
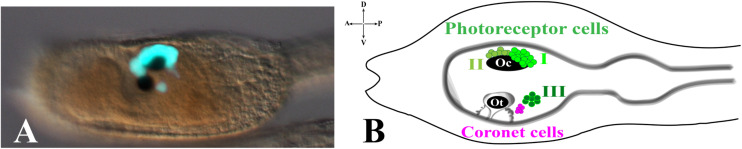
Pigmented organs in the sensory vesicle. **(A)** pArr>eGFP expression in photoreceptor territories of *Ciona* larval trunk (Yoshida et al., 2004) (photo from our lab). **(B)** Schematic representation of larval trunk. Ocellus (Oc) and otolith (Ot) pigment cells. Photoreceptor cell types I, II are associated with ocellus pigment cell, while photoreceptor cell type III is in close proximity to otolith pigment cell and coronet cells.

The ascidian larval brain (or sensory vesicle), located in the larval trunk, contains almost two thirds of the total number of the cells constituting the whole CNS (215/335). The two most evident structures present in the larval brain are the pigmented sensory organs, the otolith and the ocellus (Figure 1). The most ventral one is the otolith, made of a pigmented cell, two associated ciliated cells and two glutamatergic antennae sensory neurons. The function of the two otolith-associated ciliated cells is not known, but they could be involved in detecting movements of the otolith cell. The otolith cell is anchored to the cavity of the brain vesicle by a stalk and can move inside the cavity; furthermore, the otolith cell can change its density since its melanin granules are able to chelate metal ions. The otolith is responsible for gravity perception and pigmentation is essential for proper geotactic behavior (Tsuda et al., 2003; Sakurai et al., 2004; Jiang et al., 2005). The two antenna cells are able to detect gravity and project their axons to eleven interneurons located in the posterior brain that in turn is connected, through the neck, to the motor ganglion (Ryan et al., 2016). Ascidian larvae exhibit conserved phototactic and geotactic behaviors. Free-swimming larvae show geonegative response for most of the larval dispersal period until shortly before settlement, when they start to swim toward gravity to attach on a substrate and start metamorphosis. It has been demonstrated that pigmented cells of the ocellus and otolith are necessary for sensing light and gravity, respectively, so guiding the swimming behaviour before settlement (Tsuda et al., 2003; Sakurai et al., 2004).

The ocellus is a multicellular structure necessary for light perception and is located in the sensory vesicle on the dorsal-right side. Ocellus is made of a cup-shaped pigment cell, 3 lens cells (that are not homologous of vertebrates lens) and is associated with almost 30 photoreceptor cells, gathered into 3 groups, based on typical morphological characteristics: Group I (PR-I), Group II (PR-II), and Group III (PR-III) photoreceptor cells (Horie et al., 2008; Figure 1). The Group I, made of 18–23 cells, and the Group II, made of 8–11 cells, are both in proximity of the ocellus pigment cell. The Group I is in close contact with the pigment cell and the outer segment of each cell is arranged in rows inside the pigment cup. Their function is to perceive the light directed to the cup through the three lens cells, while the pigment cell protects them from light coming from other directions. The Group II photoreceptors are in more anterior position and outside of the pigment cup, without being protected by the pigment cell, thus sensing the light from any direction. These morphological differences and location did suggest that Groups I and II have distinct functions (see the paragraph Visuomotor and gravitaxis circuits). The Group III consists of 6–7 photoreceptor cells located far from the ocellus pigment cell and closer to the otolith. The shape of these photoreceptor cells is circular, and their outer segments are exposed into the lumen of the sensory vesicle. The Group III alone is not sufficient to evoke a photoresponse behavior in *Ciona* larvae, as demonstrated by laser ablation experiments of Groups I and II photoreceptor cells (Horie et al., 2008). This, however, does not exclude the contribution of Group III to the process. Group III photoreceptor cells differentiate later, compared to Group I and II, as revealed by immunostaining experiments with anti-Arrestin and anti-Opsin1 antibodies. The staining with both antibodies persists during early stages of metamorphosis, thus suggesting the potential function of Group III photoreceptor cells in the late larval-early metamorphosis stages. Close to the Group III is present a cluster of cells, called coronet cells, able to produce dopamine (DA), whose function is still unknown (Figure 1B; Eakin and Kuda, 1971; Nicol and Meinertzhagen, 1991; Moret et al., 2005). Some authors (Eakin and Kuda, 1971) suggested that these cells could be involved in pressure detection (hence the name of pressure organ), while others (Razy-Krajka et al., 2012) have speculated their implication in the photic response; however, in both cases these hypotheses need to be supported by experimental data.

It is intriguing to note that *Ciona* ocellus and vertebrate eyes share specific features of photo-transduction, which led to suppose a common origin of both structures (Kusakabe et al., 2001; Sato and Yamamoto, 2001). In particular, *Ciona* photoreceptors are hyperpolarizing and ciliary, like those of vertebrates (Gorman et al., 1971) and use visual opsins, G-protein coupled receptors, and visual arrestins, small proteins needed to regulate opsin signal transduction, during photo-transduction process, as their vertebrate counterpart (Arshavsky et al., 2002; Blomhoff and Blomhoff, 2006). Notably, both genes, precisely *Opsin1* (three *Opsin* genes are present in *Ciona* genome) and *Arrestin* (one *Arrestin* gene is present in *Ciona* genome), are expressed in the three groups of *Ciona* photoreceptor cells (Kusakabe et al., 2001; Horie et al., 2002, 2008; Nakashima et al., 2003).

## Photoreceptor Cell Lineage in *Ciona*

**FIGURE 2:**
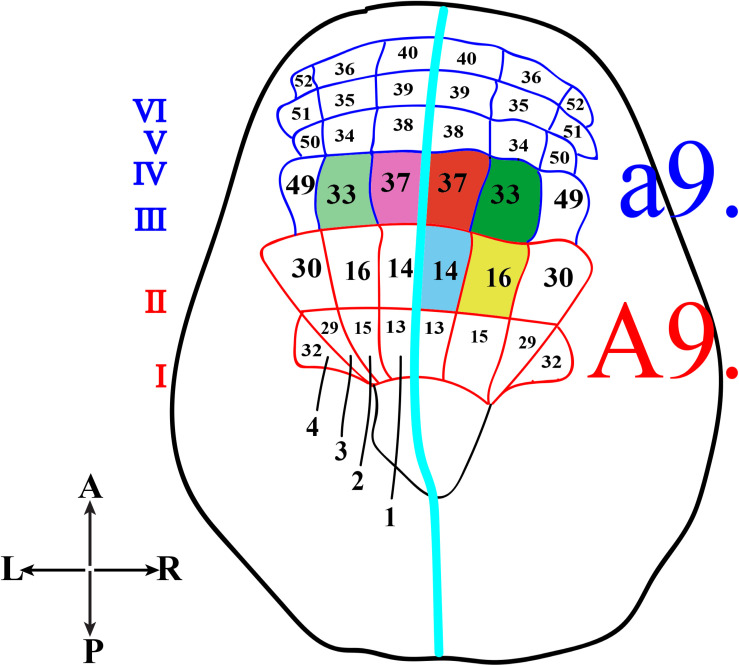
Schematic representation of *Ciona* neural plate stage. Neural plate, dorsal view, with 4 columns (1–4) and six rows (I-VI). The turquoise line separates the two bilaterally symmetrical blastomere pairs. The former a9-lineage photoreceptor cell precursors, right a9.33 and a9.37, are in green and red, respectively, while the supposed precursors of coronet cells, left a9.33 and a9.37, are in light green and light pink. The current A9-lineage Group I and II (A9.14) photoreceptor cell precursors is indicated in blue and Group III (A9.16) photoreceptor cell precursors in yellow.

In *Ciona*, many developmental processes have been defined thanks to the precise outline of the cell lineages of most tissues and organs. In this regard, the most striking example is represented by the CNS precursors that, since the late gastrula stage (neural plate), are structured like a grid, formed by eight columns (two bilaterally symmetrical four columns) and six rows (I-VI) (Figure 2). In particular, the posterior rows I and II, formed by the anterior vegetal A-line lineage blastomeres, contains the precursors of the caudal nerve cord, motor ganglion and posterior sensory vesicle of the larvae (Imai et al., 2009). The rows III-VI, which include the anterior ectodermal a-line lineage, will form the anterior nervous system. In detail, the anterior sensory vesicle is formed by descendants of row III a-line blastomeres, cells of row IV contribute to the stomodeum as well as the anterior brain, while the progeny of rows V and VI blastomeres contributes to the palps as well as to the epidermal sensory neurons in the trunk.

Concerning the photoreceptor cells, their precursors have long been identified in the right a9.33 and a9.37 blastomeres (Figure 2, green and red, respectively), while the precursors of the coronet cells were first thought to be the left a9.33 and a9.37 blastomeres (Figure 2, light green and pink, respectively) (Nicol and Meinertzhagen, 1991; Cole and Meinertzhagen, 2004).

However, the whole scenario, more recently, has been completely changed (Gainous et al., 2015; Oonuma et al., 2016), and the precursors of photoreceptor cells have been recognized to be in more posterior regions of the neural plate, the medial regions of row II. All this was achieved by labeling specific blastomeres of “non-dechorionated” embryos to trace the developmental fates of each neural plate cell, from the late gastrula up to the larval stage. In these experiments, the authors used Kaede photoactivatable reporter downstream from the regulatory regions of *Dmrt1* gene, specific for a-lineage (Wagner and Levine, 2012), and *FoxB* gene (Imai et al., 2004), specific for A-lineage, since the 32–64 cell stage. Kaede photoconverted larvae were then analyzed by co-immunostaining with anti-Arrestin and anti-Opsin1 antibodies. In these experiments, the chorion was not removed in order to avoid any aberrant left-right asymmetry (Shimeld and Levin, 2006), often detected in “dechorionated” embryos, which in turn could alter the tracing of the correct cell lineage. The results of this study indicated that Group I, Group II and Group III photoreceptor cells originate from the A-lineage blastomeres and, specifically, those of Group I and Group II are the descendants of the right A9.14 (Figure 2, blue) cell and those of Group III derive from the right A9.16 cell (Figure 2, yellow).

## Visuomotor and Gravitaxis Circuits

Recently, the visuomotor responses of *Ciona* larva to rapid light dimming (or shadow response) and negative phototaxis have been the subject of a detailed study (Salas et al., 2018). The authors demonstrated that *Ciona* larvae tend to escape from the light and exhibit a negative phototaxis, which depends on the intensity of the illumination and is characterized by sustained directional swims. That is: lower illumination/reduced negative phototaxis; higher illumination/increased negative phototaxis. The negative phototaxis is “age-dependent”: young larvae (20.5 hpf at 18°C) lacked this behavior but were able to respond to dimming light; older larvae (from 23 hpf at 18°C) showed instead both behaviors (phototaxis and shadow response) that resulted to be quantitatively and qualitatively different. Indeed, the swims evoked by light dimming were more tortuous compared to the swims evoked by directional light (negative phototaxis) and the dimming response appeared independent of light direction. Interestingly, the albino mutant pristine (*prs*), which does not contain pigmented cells in the brain vesicle but has photoreceptors, showed no phototactic behavior, supporting previous assertions that phototaxis is linked to Group I photoreceptor cells associated to a pigmented cell (Mast, 1921; Svane and Young, 1989; Jiang et al., 2005). *Prs* larvae, however, showed an elevated shadow response and a lower tortuosity of the resulting swims, compared to control larvae, thus indicating their positive photoresponse.

*Ciona* connectome, recently mapped, revealed the smallest CNS known in any chordate, with only 177 neurons, distributed in the three structures (Ryan et al., 2016). These neurons can be split into at least 25 types and each of them has, on average, 49 synapses with other cells. Thus, despite the small number of neurons, the neuron network shows a substantial complexity. These minimal and specific neural circuits, simple versions of the intricate networks of vertebrates, offer a unique opportunity to describe basal features of chordate CNS and to study the influence of each single neuron on the behavior of *Ciona* larva.

**FIGURE 3:**
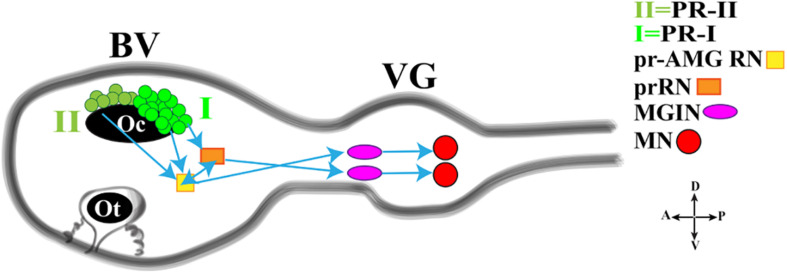
Cartoon of a *Ciona* tadpole larva Central Nervous System showing the minimal visuomotor circuit. D., dorsal; V., ventral; A., anterior; P., posterior; PR-II, photoreceptor group II; PR-I, photoreceptor group I; pr-AMG RN, Photoreceptor Ascending Motor Ganglion Relay Neuron; prRN, photoreceptor Relay Neuron; MGIN, Motor Ganglion InterNeuron; MN, Motor Neuron. BV, Brain Vesicle; VG, Visceral Ganglion. Adapted from Kourakis et al. (2019).

In this regard, a fine study depicted the minimal circuit in which the activation of motor neurons is linked to both the Group I and Group II photoreceptor cells (Figure 3; Ryan et al., 2016). Both Groups extend synapses in the posterior Brain Vesicle (BV). Group I (PR-I) transmits inputs to photoreceptor Relay Neurons (prRN) which in turn connects to the paired right/left Motor Ganglion (MGIN) interneurons and then to motor neurons (MN, five on each side). Group I (PR-I), together with Group II (PR-II), connects also to the photoreceptor Ascending MG RNs (pr-AMG RN) which are so-named because they, unlike the prRNs, receive input from the Ascending MG peripheral interneurons (AMG neurons; not shown in Figure 3). Notably, the pr-AMG RNs and the prRNs are also highly interconnected. Like the prRNs, the pr-AMG RNs extend synapses to the left and right Motor Ganglion InterNeurons (MGIN) which in turn are connected to the paired right and left motor neurons (MN). Thus, a complete visuomotor circuit, moving from photoreceptors up to the muscle target cells, is present in *Ciona* connectome (Figure 3).

This simple circuit has been recently connected with neurotransmitter use and behavioral observations, thanks to a study by Kourakis and his collaborators (Kourakis et al., 2019). The authors, by fine and elegant experiments, inferred a model in which *Ciona* exploits the PR-Is to sense the direction of light and PR-IIs to perceive the changes in ambient light. The PR-I circuit uses glutamate, is excitatory and projects, as previously reported, to both cholinergic prRNs (expressing the glutamate AMPA receptor (AMPAR)) (Okamura et al., 2005) and GABAergic pr-AMG RNs (Figure 3), which in turn synapses onto the cholinergic MGINs then connected to MNs. Notably, the interconnection between pr-AMG RNs and prRNs suggests that these clusters are not simple conveyors of information, from photoreceptor cells to motor neurons, but they can play a primary role in visual processing.

The PR-II circuit uses mainly GABA and synapses onto the GABAergic pr-AMG RNs, thus indicating that the PR-II output to the pr-AMG RNs is predominantly inhibitory (Figure 3). Notably, some PR-IIs co-express VGAT and VGLUT and this, although speculative, could be related to the need of fine tuning excitatory/inhibitory balance.

**FIGURE 4:**
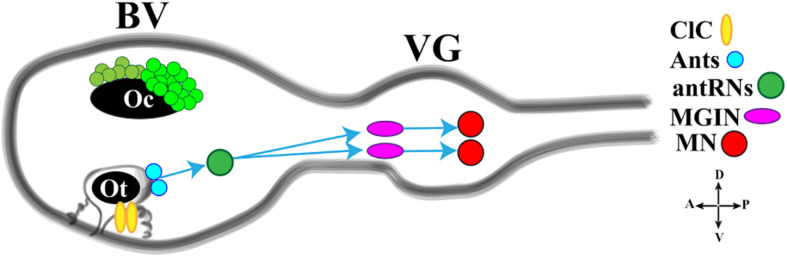
Cartoon of a *Ciona* tadpole larva Central Nervous System showing the minimal gravitaxis circuit. D., dorsal; V., ventral; A., anterior; P., posterior; ClC, ciliated cells; Ants: glutamatergic antennae sensory neurons; antRNS, GABAergic antenna Relay Neurons; MGIN, Motor Ganglion Inter Neuron; MN, Motor Neuron. BV, Brain Vesicle; VG, Visceral Ganglion. Adapted from Kourakis et al. (2019) and Bostwick et al. (2020).

The light response is further integrated with the gravity response to generate a complex behavior that permits the rapid reorientation of larvae in response to dimming. Gravitaxis in *Ciona* is controlled by the sensory otolith and the minimal gravitaxis circuit, leading to motor neurons, is made up of the otolith cell connected to the two glutamatergic antenna cells (Ants) which synapse onto the GABAergic antenna relay neurons (antRNs) asymmetrically connected to the right cholinergic MGINs and in turn to MNs (Figure 4).

Recently, Bostwick et al. (2020) investigated the neural circuits of gravitaxis in *Ciona* larvae using connectomic and neurotransmitter data, coupled with sophisticated behavioral assays. To sum up, the authors inferred that the gravitaxis circuit induces curved or tumbling (asymmetric) swimming when the light is off and the larva is head down or sideways, while, when the larva is facing upward, this circuit is almost inactive. The gravitaxis appears actively repressed until light off and the repression state is supported by the intersection between gravitaxis and photoreceptor Group II. As previously reported, the PR-II output to the pr-AMG RNs is predominantly inhibitory. Interestingly, pr-AMG RNs synaptically contact the antRNs, which are targets of the gravitaxis circuit in the posterior BV. These synaptic contacts thus result in a plausible circuit model in which inhibitory input from PR-II suppresses gravitaxis until dimming. Once the light is off, the inhibition is released, on both MGINs and antRNs, and larva initiates the asymmetric swimming. The gravitaxis circuit most probably is involved in modulating swims, by inducing curved and tumbling movements thanks to the asymmetric connections to motoneurons. Notably, the gravitaxis circuit seems to maturate during larval growth between 21 and 25 hpf.

Collectively these studies demonstrate the power of genetics, combined with behavioral analyses and connectomic data, to track down relevant functional information.

## Molecular Mechanisms Controlling Photoreceptors Differentiation

*Rx* (*retinal homeobox*)/*Rax* (retina and anterior neural fold homeobox) genes play pivotal roles in eye formation in vertebrates, since knockout of *Rx* genes results in the absence of eyes (Bailey et al., 2004). At molecular levels knockdown of *Rx* causes a reduction in the expression of the genes encoding *Arrestin* and *Rhodopsin* (Pan et al., 2010). A study on *Ciona* indicated that the gene orthologous to *Rx* exerts almost the same functions as its vertebrate counterpart. Loss of function experiments showed, indeed, that larvae lacking *Rx* do not develop photoreceptor cells, as revealed by reduced or absent expression of *Arrestin* and *Opsin* genes, and are unable to respond to light stimuli variations (D’Aniello et al., 2006). However, this study suggested that *Rx* is expressed in a-lineage cells at the tailbud stage and not in the A-lineage from which the photoreceptor cells develop, as recently assessed. To resolve this issue, the expression profile of *Rx* in *Ciona* has been recently reanalyzed, with the aim to reveal the possible presence of *Rx* in the A-lineage and substantiate its role in photoreceptor cells formation (Oonuma and Kusakabe, 2019). The authors confirmed that the expression of *Rx* starts in the a-lineage at the early tailbud stage, as previously demonstrated, but they revealed that, from the middle-late tailbud stage (13th generation cells), *Rx* expression starts in the most anterior A-line blastomeres derived from the right A9.14 cell. During next cell divisions, the *Rx* staining expands posteriorly to the whole descendants of the right A9.14 cells, while continuing to be expressed, anteriorly, in the right a-lineage descendants. Thus, the detection of *Rx* in the A9.14 lineage fits now with its involvement in photoreceptor cells differentiation.

Concerning *Rx* regulation, the authors speculated that Homeodomain and Sox Binding Sites are important for the activation of *Ciona Rx* in photoreceptor lineage, while *Onecut*, previously demonstrated to be involved in *Rx* expression (D’Aniello et al., 2011), may regulate *Rx* in the a-lineage cells, but not in the A-lineage cells for two main reasons. *Onecut* expression has not been detected so far in the A-lineage photoreceptor progenitor cells and mutations affecting the Homeodomain Binding sites (BSs), but not Onecut BSs, in the proximal enhancer region, significantly reduced transgene activity in the A-lineage cells. Thus, it appears that a combined action of activators and repressors is responsible for proper expression of *Rx* in photoreceptor lineage. Homeodomain transcription factors seem to be required for *Rx* activation, while members of *Sox* family could be involved in the repression of ectopic *Rx* expression. However, further studies are required to verify the effective role of these candidate genes in *Rx* regulation and to elucidate the detailed mechanism, acting upstream from *Rx*, controlling early specification of photoreceptor cell lineages.

## Gene Regulatory Network Underlying Pigmented Cells Specification

**FIGURE 5:**
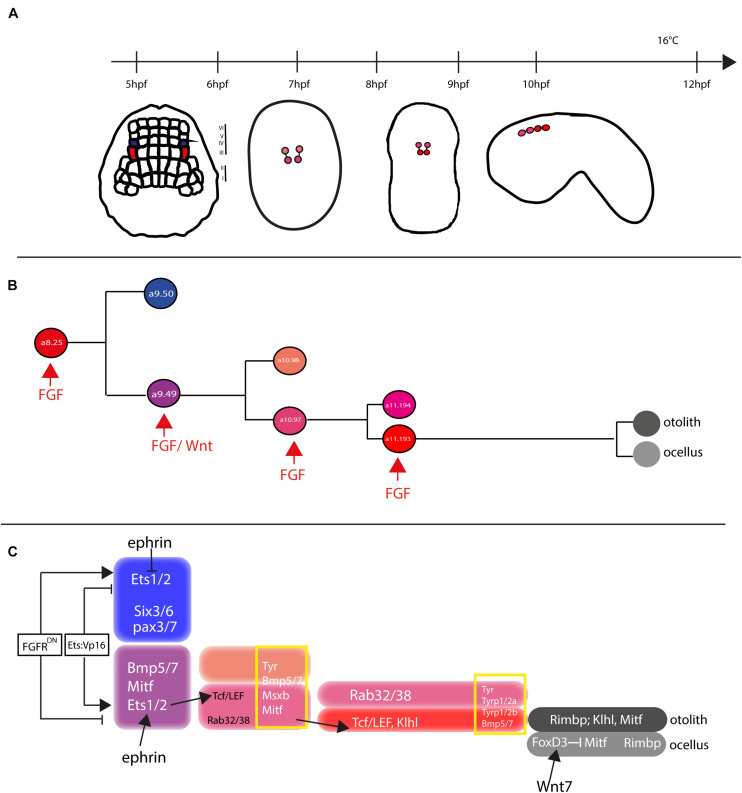
Otolith/ocellus cell fate in *Ciona robusta*. **(A)** Summary of pigmented cell lineage development within CNS, with the developmental stages (hours post fertilization, hpf) indicated the timeline [from Racioppi et al. (2014)]. **(B)** Fate determination of otolith and ocellus precursors from a9.49 blastomeres, under the control of FGF [adapted from Racioppi et al. (2014)]. **(C)** Reconstruction of genes involved in controlling otolith/ocellus determination. Adapted from Racioppi et al. (2014).

In both *Ciona* and *Halocynthia roretzi*, cell lineage of pigmented cells becomes directed toward this fate, starting from gastrula stage, in two bilateral symmetric blastomeres, the a8.25 pairs (Nishida, 1987; Cole and Meinertzhagen, 2004). Then, the a8.25 cells divide once giving rise to the a9.49 and a9.50 cell pairs and, as the development proceeds, the a9.50s (positioned in the Row IV of the neural plate) will give rise to the anterior part of the CNS, while the a9.49s (located in the Row III) will retain the fate of pigment cells (Figure 5A). Both a9.49s express *Tyrosinase (Tyr)* and *Tyrosinase related protein genes* (*Tyrp*) that are essential for melanogenesis (Tief et al., 1996; Caracciolo et al., 1997; Esposito et al., 2012; Haupaix et al., 2014; Racioppi et al., 2014). A further division of the a9.49s cell pairs, at the mid-neurula stage, give rise to the a10.97s and a10.98s pairs, which after a further division, at the early tailbud stage, will form eight post-mitotic cells (Haupaix et al., 2014; Racioppi et al., 2014) that, according to cell nomenclature described in Cole and Meinertzhagen (Cole and Meinertzhagen, 2004), are named a11.193/a11.194 and a11.195/a11.196 pairs, respectively. The eight Pigment Cell Precursor (PCP) migrate and intercalate, during neural tube closure, aligning in a single row, along the anterior-posterior axis. Among them, the fate of pigmented cells remains restricted to the a11.193 pair and the final choice among ocellus or otolith pigment cell fate is based on the anterior-posterior position of these cells. The most anterior cell migrates inside the sensory vesicle and develops as otolith pigmented cell, while the posterior one will form the ocellus pigment cell (Nishida and Satoh, 1989; Darras and Nishida, 2001).

Pigment cell precursor formation represents one of the first example of inducible cell fate in *Ciona*. It has been demonstrated that FGF signal is one of the actors responsible for this induction. Indeed, at the gastrula stage, the a9.49 cells, in row III, are both located in a position close to blastomeres of row II expressing the signaling factors Ci-fgf8/17/18 and Ci-fgf9/16/20. Moreover, the known downstream effector of FGF signaling, the transcription factor Ci-ERK1/2, is present in its activated form (phosphorylated) in the cells of the neural plate row III, including the a9.49s pigment cell precursors (Hudson et al., 2007; Racioppi et al., 2014).

The requirement of the FGF signaling for proper specification of the pigment cells in *Ciona* sensory organs has been demonstrated in several elegant experiments (Hudson et al., 2007; Squarzoni et al., 2011; Racioppi et al., 2014). FGF signaling, via the MAPK/ERK cascade and the activation of Ets1/2 transcription factor, is required for pigment cell specification in *Ciona*. Ets1/2 transcripts are present in the a9.49 cells and are inherited by their descendants, a10.97 and a10.98 blastomere pairs. Intriguingly, morphogenetic rearrangements put the a10.98 blastomeres more distant from FGF sources, in a rostral position (Figure 5B), while the a10.97s remains in close proximity of row II cells producing FGF secreted factors. As it is well known, FGF molecules are able to extend their function at short range from the target cells (Tassy et al., 2006), hence Ets1/2 factor results activated (phosphorylated) solely in the a10.97 blastomeres and able to initiate the transcription of the genes necessary for pigment cells specification. Thus, the key event in the cascade is just the activation, through phosphorylation, of Ets1/2. Differential activation of ERK1/2 in the a9.49 cell pairs is related to MAPK/ERK phosphorylation cascade and finely regulated by the presence of the Eph/ephrin signal, which has been shown to act, during gastrulation, by limiting the activation of ERK1/2 factor into the a10.97 cell pairs (Hudson et al., 2007). Indeed, interference with the Eph/ephrin signal results in extranumerary pigment cells at the larval stage and ectopic expression of *Trp* in a10.98 cells (Haupaix et al., 2014). Collectively, these data point to a fundamental role played by FGF/MAPK/ERK/Ets signaling in pigment cells specification.

Another relevant factor involved in pigment cells formation in *Ciona* is Tcf, a downstream effector of Wnt signaling (Molenaar et al., 1996). Notably, Ciona *Tcf* is transcriptionally regulated by FGF signaling, through direct control of Ets1/2 factor, and this interaction makes the a9.49 pairs “competent” to respond to Wnt signal (Squarzoni et al., 2011). The crosstalk of two signaling cascades at the transcriptional level represent a new way to study how different inputs can intertwine to control the specificity of cellular responses.

Further data on pigment cell differentiation have been obtained employing lineage-specific transcription profiling of PCP upon interference with FGF signaling. Our group demonstrated that FGF signaling induces pigment cell identity in the blastomeres that, in absence of this signal, would adopt an anterior neural fate (Racioppi et al., 2014). Among the genes identified in this analysis, *Rab32/38*, a small GTPase involved in molecular trafficking, has been studied and demonstrated, by mutational analyses, to play a role in the pigmentation of *Ciona* larval sensory organs (Racioppi et al., 2014, 2019b).

In *H. roretzi*, a further cascade involved in the pigmentation program is the BMP (Bone Morphogenetic Protein) signaling. The authors demonstrated that one or no pigment cells are formed in the larval sensory vesicle by overexpressing *BMPb* (Miya et al., 1997). When pigmented cells precursors are aligned along the dorsal neural tube, BMP signal, in antagonism with Chordin, that is present in the cell located just posterior to the PCPs, is responsible for the ocellus/otolith choice (Darras and Nishida, 2001).

Differently from *H. roretzi*, BMP/Chordin antagonism does not seem to be involved in pigment cells fate choice in *Ciona*. Data from Abitua and collaborators indicated a role by Wnt signaling in *Ciona* otolith vs. ocellus differentiation. The authors demonstrated that, at the tailbud stage, the ocellus precursor (a11.193) expresses *Tcf* gene (Abitua et al., 2012) and that *Wnt7* (one of the Wnt genes present in *Ciona* genome) is expressed, along the dorsal midline, in the blastomere just behind the ocellus precursor. If Wnt7 is misexpressed in more anterior position, both a11.193 PCP blastomeres give rise to ocelli. On the other hand, interference with *Tcf* in PCP blastomeres induces differentiation of otolith in both 11.193s.

Furthermore, a suppressive role for the transcription factor *FoxD* has been identified. *FoxD*, which is specifically expressed in ocellus precursor at the tailbud stage, exerts its function by blocking the expression of *Mitf* transcription factor in this cell, thus attenuating the formation of melanin granules and leading to the formation of the less pigmented ocellus. On the other hand, *Mitf* expression, not attenuated by the presence of FoxD factor, leads to the sustained formation of melanin granules in the densely pigmented otolith.

Taken together, these data suggest a possible gene regulatory network (GNR) responsible for the differentiation of *Ciona* otolith and ocellus pigment cells (Figure 5C) in which *Wnt7* activates *FoxD* in the ocellus precursor, which in turn suppresses *Mitf* expression in this cell (Abitua et al., 2012; Figure 5C).

Recently, a new player in the specification of PCPs, *Klhl21/30*, has been identified. This gene, belonging to the *Kelch* family of genes, has been shown to be present in a dynamic pattern in *Ciona* PCPs, starting to be expressed in both the a10.49s and then becoming restricted to the otolith precursor at the tailbud stage. To place this gene inside the GRN of the PCPs, its regulatory region has been studied. The data demonstrated that *Klhl21/30* minimal key *cis-*regulatory element, able to drive its expression specifically in *Ciona* otolith, is controlled by Mitf, Msx, and Dmrt transcription factors, that work synergically to control the specific expression of the gene (Coppola et al., 2020).

## Sensory Organs in Molgulidae: A Special Case

Despite the conservation in the general organization of the body plan of the swimming ascidian larvae, some species have developed an anural larval stage (Lacaze-Duthiers and de Lacaze-Duthiers, 1858; Berrill and Watson, 1931; Jeffery and Swalla, 1990).

About 20 species, among tunicates, present this type of larva and most of them belong to the Molgulidae family, where tail loss has occurred, independently, multiple times (Jeffery and Swalla, 1990; Huber et al., 2000). The reason why the molgula have developed a larval stage lacking some of the chordate distinctive characteristics is still not clear. Some authors suggested that the specific habitats, where flat sand is predominant and to which these species are adapted, could be at the basis of these changes, because in these natural conditions the dispersal phase is not useful and so dispensable (Huber et al., 2000).

Together with tail, anural larvae have lost most of the characteristics more directly associated with swimming, as the capability to form tail muscles (Whittaker, 1979; Swalla and Jeffery, 1990, 1992; Bates and Mallett, 1991; Bates, 1995; Tagawa et al., 1997) and this is linked to the inactivation, by pseudogenization, of genes like muscle actin, as demonstrated in *Molgula occulta* (Kusakabe et al., 1996; Jeffery et al., 1999).

Regarding sensory organs, it has been described that most of the molgulidae larvae present only the otolith, while the ocellus pigmented organ is absent, thus becoming unable to respond to light. In other species, like *Molgula occulta*, both pigmented organs are absent (Berrill and Watson, 1931) or vestigial (Swalla and Jeffery, 1992) and this loss has been associated with the absence of functional genes coding for *Tyrosinase* and *Tyrosinase related proteins* (*Tyrp*) (Racioppi et al., 2017). As an example, in *M. occulta* these genes show several mutations, like bases insertion or deletion, leading to the formation of premature stop codons into the mRNA, which becomes unable to code for functional enzymes (Racioppi et al., 2017).

These mutations are most probably still accumulating in *Molgula* genome, due to a loss of selective pressure on genes responsible for the pigmented organs formation (Racioppi et al., 2017).

## New Approaches for the Study of Ascidians Sensory Organs

Developmental biology approaches take great advantages on the interference with a gene expression (silence or overexpression) to understand its function during development of a specific cell lineage in model organisms. During the past two decades a wide range of genetic tools have been developed and, at the same time, a series of new model organisms have established themselves as good models for the study of developmental mechanisms, physiology and behavior.

## CRISPR/Cas

Most of the methods used to modify a specific sequence of DNA rely on the principle of site-specific DNA recognition by oligonucleotides, small molecules, or self-splicing introns, such as zinc finger nucleases (ZFNs) and transcription activator-like effector nucleases (TALENs). Recently, CRISPR/Cas9 technique has been shown to function, with high fidelity and efficiency, in *Ciona* (Stolfi et al., 2014; Gandhi et al., 2017). This technique, exploited by the prokaryotic adaptive immune system, has been modified for the use in eukaryotic cells. CRISPR/cas9 technology is based on the use of an oligonucleotide, called short guide RNA (sgRNA), with a sequence of 20 nucleotides identical to the target DNA sequence, and an RNA mediated nuclease Cas9. The interaction of the sgRNA with its target genomic DNA sequence guides the Cas9 nuclease to this site. Cas9 unwind the DNA and cleave both strands. During the resulting repair mechanism, by homologous recombination or non-homologous end joining, insertions and/or deletions can accumulate at the target site, thus creating a mutation in the original sequence of the gene and its transcript (Jinek et al., 2012). In *Ciona* the embryos are transfected by electroporation method, allowing tissue-specific disruption of the gene of interest. Fertilized embryos are electroporated at one-cell stage with two plasmids, one driving the zygotic expression of Cas9 protein under a lineage specific promoter of choice, the other one with the selected sgRNAs under U6 ubiquitous promoter (Nishiyama and Fujiwara, 2008; Stolfi et al., 2014; Gandhi et al., 2017). The phenotypes are normally observed in F0 generation, allowing a very rapid analysis of the mutated larvae. The disadvantage of this technique in this model organism is related to the difficulties in obtaining transgenic strains of *Ciona* in order to study the mutations obtained by gene editing in the F1 generation. However, the phenotypes in F0 generation can be exploited as a powerful tool to investigate tissue-specific functions of a gene during development. In this regard, CRISPR/Cas9 technology has been used in *Ciona* to study some aspects of the Central and Peripheral nervous system development (Stolfi et al., 2014). As an example, targeted interference with FGF signaling by CRISPR/Cas9 has been instrumental to study some aspects of CNS development by using Bipolar Tail Neuron (BTNs) as an accessible model system for neurogenesis (Kim et al., 2020). Regarding cell fate determination of the otolith and ocellus in *Ciona*, preliminary results have been obtained studying, by CRISPR/Cas9 technique, *Mitf* gene, a key regulator of melanocyte development and melanoma in vertebrates (Levy et al., 2006). The study revealed that *Mitf* is a fundamental transcription factor involved in KLHL *21/30* (a specific marker of otolith) expression in *Ciona* (Coppola et al., 2020). Further studies, using transcriptomic approaches on *Mitf* mutated embryos, will be useful to study the gene regulatory network involved in pigmented cells differentiation.

## scRNAseq

The simple organization and morphology of ascidian embryos highlights the crucial role of cell lineage during animal development. These characteristics, together with the advance in recent techniques, as single cell sequencing (scRNAseq), give the opportunity to investigate the transcriptome of a specific cell population. The use of scRNAseq technique is expanding in many model organisms including *Ciona*, where encouraging preliminary studies have been carried out. Extensive transcriptome trajectories, regulatory cascades and provisional gene networks for over 60 cell types, including pigmented cell lineage, were rebuilt by analyzing the transcriptome profiles of individual cells from gastrulation, 110 cell-stage, to larval stages (Cao et al., 2019). The use of scRNAseq has been applied to describe the transcriptome of *Ciona* different area of nervous system. In particular, for larval brain, 10 different clusters were identified and, within them, the most representative neural tissues were re-clustered, like dorsolateral brain, sensory/brain vesicle wall, epidermal neurons and ventral brain (Sharma et al., 2019). RNA sequencing allowed to explore the features of the lateral plate ectodermal of *Ciona*, which have common traits with neural plate ectoderm in vertebrates; both systems reveal similarities in the compartmentalization and regulatory program of *Six 1/2, Pax3/7* and *Msx* expression. These results support the hypothesis that the compartmentalization of lateral plate ectoderm preceded the origin of vertebrates (Horie R. et al., 2018). Moreover, the regulatory network underlying the specification of coronet cells, a class of dopaminergic neurons in *Ciona*, has been elucidated by using RNAseq technique (Horie T. et al., 2018). Single cell technology can thus be used to reconstruct the developmental patterns of the Central Nervous System. Moreover, the outputs of scRNAseq coupled with CRISPR/Cas9 technology can greatly improve our understanding about the interaction amongst the identified genes during specification and differentiation of the tissues of interest.

Although most of the processes regarding early neural development and some of the related gene regulatory pathways have been identified (Satoh et al., 2003; Sasakura et al., 2012), the results of the studies on scRNAseq of neural tissues of *Ciona* represent a great and encouraging starting point to continue using this technique to thoroughly depict the single cell transcriptome profiles, since early stages of *Ciona* embryogenesis in order to finely reconstruct, step by step, the developmental patterning of the Central Nervous System.

## ATAC–seq

Animal development is driven by changes in the gene regulatory networks, which play a key role in the evolution of the animal body plans. The ATAC–seq represents a powerful technique for the identification and study of chromatin accessibility. Applying this technique in embryos, at different developmental stages and on different cell types, permits to investigate the regulatory pathways in the regions of interest (Magri et al., 2019). ATAC-seq has been recently developed for *Ciona* to profile chromatin accessibility through transitions from mesoderm to distinct fate-restricted heart and pharyngeal muscle precursors (Racioppi et al., 2019a).

These recent and extraordinary advantages in biological techniques, together with the affirmation of the tunicate as simple chordate model organism, are growingly increasing the possibilities to shed light on the multiple aspects and functions of the genes and their regulatory pathways.

## Conclusion

In the course of evolution, increasingly intricate gene regulatory networks have been elaborated to orchestrate the specification, patterning and differentiation of diverse cell types in order to build up complex and different organisms. Ascidians, with their unique phylogenetic position, together with the simplicity of their typical chordate body plan and structures, represent for more than a century a powerful model organism to approach “simplified versions” of complex biological mechanisms. In these organisms it is possible to study gene regulatory networks, with a cellular resolution unprecedented in chordate models. Furthermore, the adaptation to this model system of the most updated technologies and high-throughput strategies for targeted loss-of-function and whole genome analyses, coupled with the use of live imaging approaches and computational methods, are more and more permitting to get important insights on developmental strategies exploited during chordate evolution. A clear example is just represented by the sensory organs, for which we are close to depict the developmental programs adopted by pigmented and photoreceptor cell lineages, at single cell level, starting from the early developmental stages, within the frame of whole CNS development. Under this perspective, the single cell transcriptomic analyses and the data already available will be instrumental to depict the set of transcripts of each lineage in order to better define the evolutionary relationships (i) of *Ciona* PR complexes with the photoreceptor organs of other chordates and (ii) of BV with vertebrate midbrain, with the aim to get further insights on the appearance and modifications of these structures in the course of evolution.

Collectively, these studies will permit to reveal the basic developmental mechanisms for forming chordate pigment and photoreceptor cells and the common/different evolutionary strategies adopted by higher vertebrates to build more complex structures.

Furthermore, the application of the most cutting-edge techniques can be extended to other biological fields besides developmental biology, as evolution and ecology, with the purpose to deepen the knowledge in the emergent discipline of Eco-evo-devo.

## Author Contributions

AP and PO: reviewing bibliography and drafting the manuscript. AS and FR: drafting and revising the manuscript. All authors contributed to the article and approved the submitted version.

## Conflict of Interest

The authors declare that the research was conducted in the absence of any commercial or financial relationships that could be construed as a potential conflict of interest.

## Publisher’s Note

All claims expressed in this article are solely those of the authors and do not necessarily represent those of their affiliated organizations, or those of the publisher, the editors and the reviewers. Any product that may be evaluated in this article, or claim that may be made by its manufacturer, is not guaranteed or endorsed by the publisher.
